# Perinatal Outcomes of Uninsured Immigrant, Refugee and Migrant Mothers and Newborns Living in Toronto, Canada

**DOI:** 10.3390/ijerph10062198

**Published:** 2013-05-31

**Authors:** Karline Wilson-Mitchell, Joanna Anneke Rummens

**Affiliations:** 1 Midwifery Education Program, Ryerson University, 350 Victoria Street, Toronto, ON M5B 2K3, Canada; 2 Community Health Systems Resource Group, The Hospital for Sick Children & Department of Psychiatry, University of Toronto; 555 University Avenue, Toronto, ON M5G 1X8, Canada; E-Mail: anneke.rummens@sickkids.ca

**Keywords:** perinatal outcomes, uninsured, immigrant, refugee, migrant, women, newborns

## Abstract

Canadian healthcare insurance is not universal for all newcomer populations. New immigrant, refugee claimant, and migrant women face various barriers to healthcare due to the lack of public health insurance coverage. This retrospective study explored the relationships between insurance status and various perinatal outcomes. Researchers examined and compared perinatal outcomes for 453 uninsured and provincially insured women who delivered at two general hospitals in the Greater Toronto Area between 2007 and 2010. Data on key perinatal health indicators were collected via chart review of hospital medical records. Comparisons were made with regional statistics and professional guidelines where available. Four-in-five uninsured pregnant women received less-than-adequate prenatal care. More than half of them received clearly inadequate prenatal care, and 6.5% received no prenatal care at all. Insurance status was also related to the type of health care provider, reason for caesarean section, neonatal resuscitation rates, and maternal length of hospital stay. Uninsured mothers experienced a higher percentage of caesarian sections due to abnormal fetal heart rates and required more neonatal resuscitations. No significant difference was found for low birth weight, preterm birth, NCIU admissions, postpartum hemorrhage, breast feeding, or intrapartum care provided.

## 1. Introduction

Various healthcare organizations have stressed the importance of adequate prenatal care as an important predictor of optimal perinatal outcomes [1,2,3,4,5]. However, many immigrant and migrant women in Canada and the United States receive less than the recommended level of care due to their lack of public health care coverage [1]. Exactly what the impact of the lack of health care insurance on perinatal outcomes may be remains a question that has been largely unexplored. 

According to the U.S. Census Bureau Insurance Coverage reports, in the year 2010, 33% of uninsured residents were foreign-born (13.17 million), 44.2% were non-citizens (9.7 million), and only 13.2% of the uninsured were native-born (35.4 million) [6]. Uninsured status is thus highest among immigrant populations, most notably among non-citizens. Undocumented migrant women in the United States who lack prenatal care have almost four times the risk of low birth weight (LBW) babies and more than seven times the risk of preterm babies (PTB) [1]. Minority, low income, and newcomer women who have limited or no access to health care also contribute heavily to American statistics for the leading causes of maternal death: thrombotic embolism (20%), hemorrhage (17%), pre-eclampsia and eclampsia (16%), infection (13%) and cardiomyopathy (8%) [7]. 

While comparable figures are not available in Canada, new immigrant and refugee women do face social and economic barriers to healthcare within the Ontario healthcare system. Canadian citizens and landed immigrants are eligible for provincial health insurance coverage, with some exceptions. Uninsured groups include: new immigrants within the first three months of their arrival as permanent residents, refugee claimants who lack Interim Federal Health care coverage, temporary workers whose visas have expired, and visitors [8,9]. Maternity care is available and provided by family physicians (with obstetrical specialization), obstetricians and midwives. However, unlike their insured counterparts, uninsured pregnant women must pay for diagnostic tests, physician fees and hospital fees. Uninsured new immigrant, refugee, and undocumented migrant women must also navigate a patchwork of unfamiliar health services to obtain the care they need. 

Newcomer women in the Greater Toronto Area report economic barriers to access for gynecological and obstetrical care in particular [10]. Several Canadian reports describe the uninsured as having poorer perinatal outcomes in general [2,11,12], however, the impact of barriers such as lack of health insurance on health outcomes for women and their newborns still requires quantitative exploration. To date, few studies have examined the perinatal outcomes of uninsured mothers and their newborns within the Canadian context and compared them with the outcomes of their insured counterparts.

Using health records information, this study explores the relationship between insurance status on perinatal outcomes and health statistics [13,14] of insured and uninsured women who received maternity care from various providers in two general hospitals located in the eastern part of the Greater Toronto Area. A convenience sample was drawn from the records of these hospitals which serve a geographic catchment area that has a population of 1,419,745, including 935,035 immigrants and 11,915 non-residents, *i.e.*, only one-third of the population is Canadian-born [15]. We hypothesized that there was a relationship between health insurance status and perinatal outcomes of mothers and their newborns. 

## 2. Methods

Institutional Ethics Review Board approval for this study was obtained from Ryerson University and the Rouge Valley Health System, Toronto, Canada. A retrospective chart review was conducted of hospital records for mothers and newborns where births occurred between 2007 and 2010, inclusive, in two Toronto area community hospitals. Data drawn from a total of 453 charts, 175 uninsured and 278 insured, were analysed to compare perinatal outcomes for insured and uninsured mothers.

### 2.1. Insurance Inclusion and Exclusion Criteria

Health insurance status can be easily identified from admission records and documentation in each health record using hospital payment codes. For the purposes of this research, “uninsured” was operationalized as referring to those pregnant women seeking care who did not have provincial health care insurance under the Ontario Health Insurance Plan (OHIP), federal health care benefits under the Interim Federal Health Benefit (IFHB) programme, or private health insurance, *i.e.*, no health insurance of any kind. At the time of the study these included: (i) new immigrant permanent residents within the three month waiting period for provincial health care coverage, as well as those currently applying for landed immigrant status through their spouse; (ii) successful refugee claimants not covered by the Interim Federal Health Programme benefits, asylum-seekers awaiting decision regarding their refugee claim, and those whose claims have been denied; and (iii) undocumented or partially documented migrants who have outstayed a visitor’s visa or work permit, or who have entered the country through non-regular means. Health care coverage provisions for refugee claimants/asylum seekers under Canada’s Interim Federal Health Programme underwent significant changes beginning on 30 June 2013. This study predates these changes. The study’s insured population sample consisted of all pregnant women, regardless of place of birth or citizenship status, who had a valid provincial OHIP card. These included both Canadian-born and landed immigrant women, as well as temporary workers and visitors with some type of temporary OHIP coverage such as farm workers, individuals on work permits, graduate students, clergy and missionaries. The study’s uninsured population sample was generated from hospital record lists using self-pay payment codes. Three sub-groups were excluded from analysis. Refugee claimants covered for medical care under the Interim Federal Health Benefits programme were excluded because this insurance plan provides partial coverage of services. Individuals with health care coverage from other Canadian provinces or with private health insurance were excluded, as were homeless women. The latter were identified in the documentation by the admissions department, social worker, or addresses of local women’s shelters in the address fields. Of the 325 non-OHIP charts pulled, 150 were excluded for these reasons, yielding 175 uninsured files for full data extraction. 

### 2.2. Clinical Exclusion Criteria

To ensure that both the insured and uninsured groups were homogenous with respect to obstetrical or medical risk, 19 women who had pre-pregnancy or pre-existing high risk medical conditions which would have predisposed them to preterm labour, placental insufficiency, maternal complications, or high risk of adverse neonatal outcomes such as hypoxic ischemic encephalopathy, preterm birth or low birth weight [16] were excluded (e.g., uncontrolled gestational or insulin dependent diabetes, substance abuse, twin pregnancy, genetic disorders or anomalies, non-cephalic presentation, known spina bifida, substance abuse, known congenital anomalies, and known footling breech). Excluding these types of pre-existing conditions ensured that the uninsured and insured study groups used for the comparative analyses were similar with respect to parity and previous medical and obstetrical histories. Prior to making the exclusions, the study’s insured population sample of 350 OHIP records was generated randomly from the hospital’s computerized list of all OHIP patients during the four year period to avoid the clustering of births at any particular time during this period. The final study sample of 453 cases included: 175 uninsured immigrant, refugee and migrant pregnant women without medical insurance coverage of any kind, and 278 insured pregnant women with provincial Ontario Health Insurance (OHIP) coverage. 

### 2.3. Coding

Each chart in the sample was reviewed for accuracy of coding and categorization prior to data extraction, and only complete charts were retained (a few charts lacked pertinent data and were excluded for that reason). Confidentiality and privacy was maintained by assigning non-traceable research coding to replace identifying personal information. 

### 2.4. Retrieved Data and Statistical Analyses

The a priori dependent variables used in the analyses were: number of prenatal visits, provider type, caesarean section (C/S) rate, maternal complications (e.g., postpartum hemorrhage), neonatal complications (e.g., newborn resuscitation), low birth weight (LBW), small for gestational age (SGA), preterm birth (PTB), Neonatal Intensive Care Unit (NICU) admission, length of hospital stay and exclusive breastfeeding upon discharge [13,14]. The designation of small for gestational age (SGA) was calculated using Lubchenco’s Table [17] in which a weight falling less than 2 standard deviations below the mean for that gestational age is considered SGA. Data extraction of key perinatal health status indicators from client medical records was greatly facilitated using computer-assisted data capture and a prepared electronic template [18]. The health indicators selected were chosen for their relevance and ability to inform future hypothesis development and research design [19,20,21]. Care was taken to extract information only from complete records. Wherever possible, both maternal and newborn charts were reviewed to maximize accuracy and completeness [20,22,23]. 

Extracted medical records data were analyzed using SPSS version 16. Bivariate analyses were used to describe and compare medical interventions for the two study groups: frequencies, chi-squared, and non-parametric t-tests. Means and percentages were calculated to describe the health variables and test for differences between the groups for each dependent variable or factor such as smoking. Means and confidence intervals [24] were compared to provincial rates where available. Other findings were compared to existing professional standards. Multivariate analysis was considered inappropriate because most of the dependent variables are nominal (and some of them dichotomic variables) and insurance status is a dichotomic independent variable.

## 3. Results

### 3.1. Socio-Demographic Description

**Figure 1:**
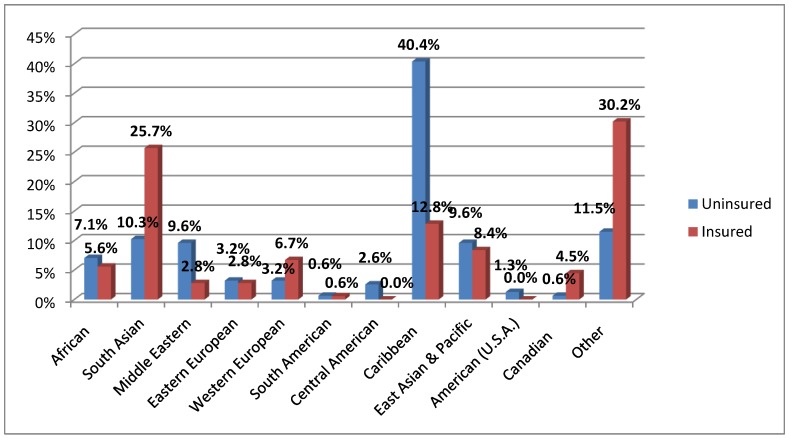
Ethnic background by insurance status (*n* = 453).

The two sample groups were similar in terms of age, parity, obstetrical risk status, and gestational age. While the mean age for both groups was 29 years, the mode for the insured was 30 years and the mode for the uninsured was 27. Although ethnic data was not available from the main data source, we know that both groups were ethnically diverse, based on analysis of the patients’ Ontario Antenatal Records, as shown in Figure 1. Matching of uninsured and insured charts on the basis of racial/ethnic background or income levels was not possible due to lack of such accurate, self-reported information in the medical charts. Ethnicity was based upon the prenatal healthcare provider’s notation on the antenatal records (loosely interpreted as the World Bank countries and regions). So for this study, interpretation of ethnicity is used for descriptive purposes only. Table 1 shows selected socio-demographic and clinical variables for the sample groups. Although the sample groups had similarly varied ethnic profiles, the uninsured group included a greater percentage of Caribbean women, whereas the insured group included a greater percentage of South Asians (the largest ethnic groups, respectively. This reflects existing immigration trends and trajectories. Indeed South Asian and Caribbean immigrants constitute two of the larger immigrant groups in the Toronto area [25]. A large group of insured charts (30.2%) indicated ethnicity as “mixed race”, “Black” or the field was left blank. These were classified under “other”. 

**Table 1:** Socio-demographic and clinical description of the sample groups (*n* = 453).

Sample Descriptor	Uninsured	Insured
Largest Ethnic group	Caribbean (40.4%)	South Asian (25.7%)
Mean Age (year)	28.6	29.3
Primigravidas	54.6%	52.5%
Community Health Centre Use Male Infant births	18.33% 46.0%	0% 53.7%
Mean Gestational Age (wk)	39	39
Community Health Centre Use	18.3%	0%
Gestational Diabetes	4.2%	3.2%
Chronic Hypertension Smoking	2.7% 1.26%	4.9% 5.95% (significantly higher)

Information regarding use of Community Health Centre (CHCs), as indicated on the antenatal records and diagnostic tests, was relevant because these provincially-funded centres provide free, well-integrated multidisciplinary services that are available to all Ontario residents regardless of citizenship status; CHCs are well designed to meet both the social and medical (non-obstetrical) needs of vulnerable groups such as the uninsured. CHC involvement provided an indication of access to community resources. Use of a CHC as a community resource was also documented in 18.3% of the uninsured records. 

### 3.2. Clinical Descriptions of the Sample Groups

Smoking was indicated in 5.9% of the insured cases and in only 1.3% of the uninsured. The insured charts also had a higher number of prenatal maternal health complications, such as mild asthma, well-controlled hypothyroidism, chronic but well-controlled hypertension or diet-controlled gestational diabetes. It is unknown how many uninsured women were under-diagnosed or undertreated for these conditions. Well-controlled gestational diabetes complicated the pregnancies of 4.2% of the insured group and 3.2% of the uninsured group. Similarly, 4.9% of insured and 2.7% of uninsured pregnant women had well controlled chronic hypertension. The numbers of hypertensive and diabetic cases were not large enough to test for statistical difference in relation to insurance status. 

### 3.3. Analysis of Dependent Variables

**Figure 2:**
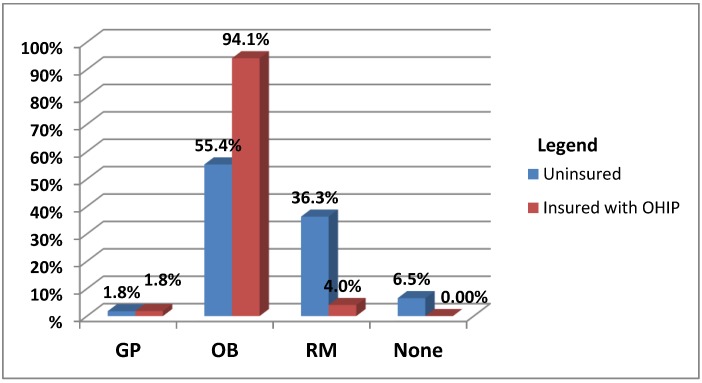
Prenatal healthcare provider type by insurance status (*n* = 453).

The null hypothesis was met for only a few of the key perinatal outcome indicators: amount of prenatal care, type of provider, reason for cesarean section, the need for neonatal resuscitation and maternal length of hospital stay. Review of all medical records revealed that prenatal care was provided by obstetricians, registered midwives and general practitioners who were primarily family doctors from CHCs who quickly transferred care to a midwife or obstetrician. The uninsured pregnant women sought the services of midwives significantly more than did the insured, 36.3% *versus* 4.0% (see Figure 2). 

This is likely due to the fact that midwifery care is funded by the provincial Ministry of Health and Long Term Care and is therefore essentially free to uninsured women who are considered “residents”. Slightly more than half of the uninsured pregnant women received the services of an obstetrician during their pregnancy, while almost all insured women did so. 

**Figure 3:**
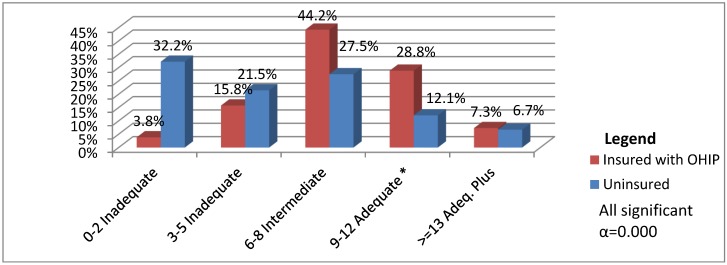
Number of Prenatal Visits by Insurance Status (*n* = 453) Adequate means ≥9–12 visits beginning by the 4th month (based upon SOGC Guidelines).

Perhaps most revealing is that 6.5% of uninsured women received no prenatal care at all. By contrast, all insured women received care. As anticipated, the number of prenatal visits reported for the uninsured group (mean = 6.04, t = −6.173, α = 0) was significantly lower, than for their insured peers (mean = 8.70). According to the Society of Obstetricians and Gynaecologists of Canada (SOGC), using these guidelines, adequate prenatal care is comprised of 9 to 12 visits starting during the fourth month of pregnancy or earlier [2,16,26]. Inadequate prenatal care can therefore be operationalized as fewer than nine visits that began after 21 weeks gestation. Using these guidelines, more than half (53.7%) of the uninsured women had received clearly inadequate prenatal care, in contrast to only one-in-five (19.6%) insured women (see Figure 3). Moreover, insured women, as a group, had comparatively higher levels of intermediate care (six to eight visits); and uninsured women had significantly higher rates of clearly inadequate care (zero to five visits). In all, four-in-five uninsured women received less than adequate prenatal health care. 

No significant differences were found between the insured and uninsured groups with respect to LBW rates, PTB rates, overall maternal complications, or breastfeeding rates (see Table 2). 

**Table 2:** Selected factors and perinatal outcomes of uninsured *versus* insured cases (*n* = 453).

	Insurance Status			
Outcomes	Uninsured (*n* = 175) 95% CI ^a^	Insured (*n* = 278) 95% CI ^a^	Statistic ^b^	Significance (α) (*P* value)
Number of Prenatal Visits	6.04 (5.40–6.68)	8.70 (8.35–9.05 )	t = 6.173	0.001
Preterm Birth (PTB) Rate	7.43% (3.54–11.31)	8.27% (5.04–11.51)	χ^2^ = 0.105	NS ^c^
Low Birth Weight (LBW)	5.71% (2.28–9.15)	7.97% (4.78–11.17)	χ^2^ = 0.827	NS ^c^
Small for Gestational Age (SGA)	7.43% (3.54–11.31)	9.35% (5.93–12.78)	χ^2^ = 0.505	NS ^c^
Length of Stay Mother (mean number of days)	1.65 (1.49–1.82)	2.32 (2.19–2.46)	t = −6.11	0.001
Length of Stay Baby (mean number of days)	2.05 (1.54–2.55)	2.14 (2.00–2.29)	t = −0.36	NS ^c^
Neonatal Resuscitations	9.71% (5.33–14.1)	4.33% (1.93–6.73)	χ^2^ = 5.174	0.023
NICU Admissions	14.37% (9.16–19.58)	15.16% (10.94–19.39)	χ^2^ = 0.053	NS ^c^
Caesarean Section (CS) Rate	26.29% (19.76–32.81)	35.61% (29.98–41.24)	χ^2^ = 4.292	0.038
Caesarean Sections for Abnormal FHT	29.17% (35% excluding RCS ^d^) (16.31–42.03)	13.27% (21.7% excluding RCS ^d^) (6.55–19.98)	χ^2^ = 5.405	0.02
Caesarean Sections for Labor Dystocia	16.67% (20% excluding RCS ^d^) (6.12–17.21)	17.35% (28.3% excluding RCS ^d^) (9.85–24.84)	χ^2^ = 0.011	NS ^c^
Postpartum Hemorrhage (PPH)	3.75% (0.81–6.69)	4.03% (1.58–6.48)	χ^2^ = 0.021	NS ^c^
Breastfeeding Rate	91.02% (86.68–95.35)	86.45% (82.39–90.51)	χ^2^ = 2.077	NS ^c^

^a^ CI for p values close to or equal zero were calculated using the procedure described by Robert Newcombe, 1998; ^b^ Chi-squared tests were used to analyze categorical variables and t-tests used for continuous variables; ^c^ NS = not significant; ^d^ RCS refers to repeat caesarean sections (both elective and planned for medical reasons).

The two groups also had similar rates of intrapartum medical interventions. Of note, both uninsured and insured groups had fairly high rates of oxytocin augmentation and cesarean section compared to provincial rates [27,28]. Oxytocin augmentation occurred more often in the insured *versus* the uninsured group; this difference, however, was not statistically significant. There was no significant difference in the rate of postpartum hemorrhage. Smoking is a risk factor for preterm birth and low birth rate [29], and the insured had a significantly higher percentage of smoking than the uninsured (5.95% *versus* 1.26%), but this did not translate into seeing a higher rate of maternal or newborn complications in the insured group. 

**Figure 4:**
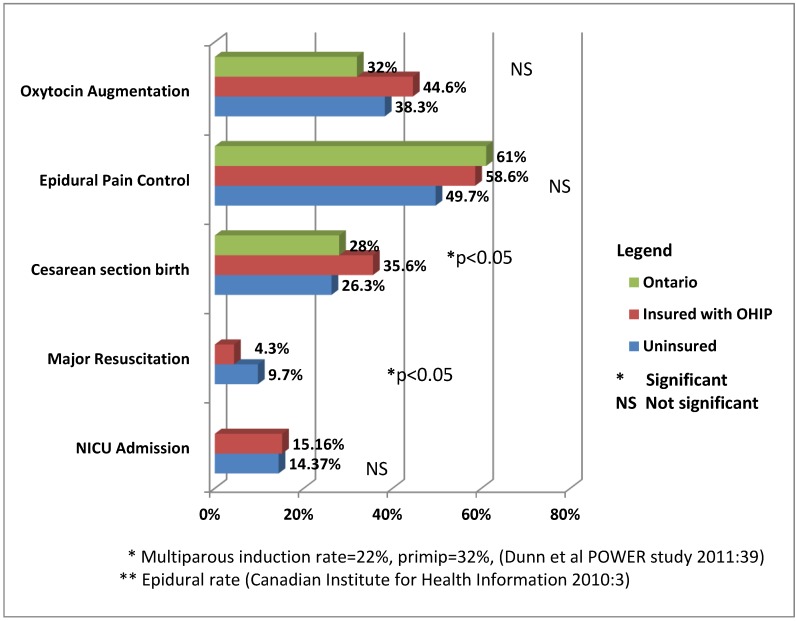
Rates of medical interventions by insurance status (*n* = 453) and in general population.

Rates of other medical interventions varied between the two groups. More caesarean sections occurred in the insured group (35.6%) than in the uninsured group (26.3% with Pearson’s χ^2^ = 4.292, α = 0.038). However, the reasons for CS differed across the two groups. When all of the planned repeat and ERCS cases were excluded from consideration, the most common reason for caesarean sections among the insured women was labor dystocia. Labor dystocia was defined as less than 2 cm of progress within 4 h or less than 1 cm descent per hour in the second stage of labor [30]. In contrast, the uninsured women had a significantly higher rate of caesarean sections due to abnormal fetal heart rate (35% *versus* 21.7%, with χ^2^ = 5.405, α = 0.020). The newborns of uninsured mothers had a significantly higher incidence (9.7% *versus* 4.3% with χ^2^= 5.174, α = 0.023) of major resuscitation involving positive pressure ventilation and/or heart compressions than did those of insured mothers; however, the rate of this intervention usually ranges between 1% and 10% in North America [31]. NICU admission rates were similar and fairly low, implying effective resuscitation (see Figure 4).

**Figure 5:**
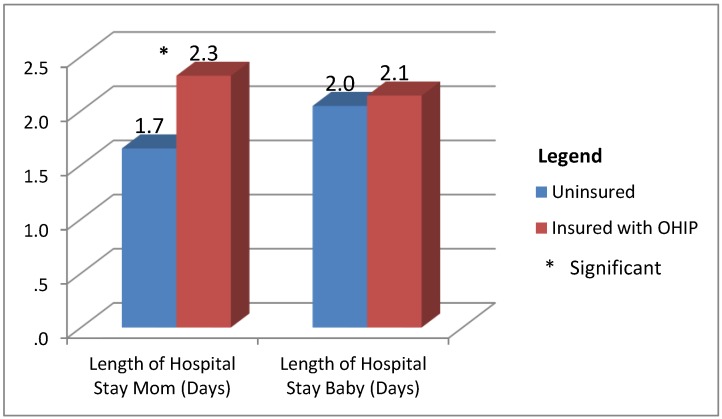
Mean length of hospital stay by insurance status (*n* = 453).

Rare events that occur in larger populations, such as meconium aspiration syndrome, major congenital anomalies, severe birth trauma, still birth and neonatal seizures, were not seen. There were two cord prolapses during the deliveries of insured cases. The length in days of hospital stay was significantly less for the uninsured mothers (1.7 *versus* 2.4 with t = −6.110, α = 0.000) than the insured mothers (Figure 5). Exclusive breastfeeding was continued at discharge by 91% of the uninsured and 86.4% of the insured upon discharge; this compares to a Canadian breastfeeding rate of 90.3% [32,33].

## 4. Discussion

In this study, health insurance status was found to be related only to the amount of prenatal care, type of health care provider, reason for caesarean section, neonatal resuscitation incidence, and maternal length of hospital stay. In contrast to findings elsewhere in the research literature, there was no significant difference in LBW rates and PTB rates of newborns delivered by insured *versus* uninsured women. Nor was lack of insurance associated with the amount or type of medical intervention provided. In fact, both the insured and uninsured groups had high rates of expensive and human resource-intensive medical interventions such as oxytocin augmentation, with the attendant financial hardships for the uninsured women who bear the associated costs themselves. Both insured and uninsured pregnant women achieved positive neonatal outcomes. It is possible that the overall epidural rates were lower than the provincial (Ontario) norm because uninsured midwifery clients tend to avoid epidural use; information on this, however, was unavailable. Further research should examine interventions across different types of prenatal and/or intrapartum care providers to explore the impact of various types of intrapartum management models on labor progress. 

Prenatal care is considered a significant clinical intervention that improves perinatal outcomes [1,2,3,4,5]. More than half of the uninsured women sampled had received definitely inadequate prenatal care, and as little as eighty percent received less-than-adequate care, as defined by the recommended number of prenatal visits. However, the Kessner Adequacy of Prenatal Care Index is limited in that it only measures quantity and not quality of prenatal care [26]. Utilization of a multifactorial assessment model that considers the role and effects of social determinants of health during the prenatal period is equally important. Although the insured women in this study had comparatively higher levels of intermediate care, both sample groups may have been affected by the type and availability of maternity care providers in Ontario. The research literature describes an increasing number of women who are late to receive prenatal care or must travel great distances for care due to the lack of an available provider in their community [34]. Similarly, uninsured pregnant women may be differentially affected by the limited number of midwives available to provide services. Future research that explores relationships between the content, quality and availability of prenatal care and key perinatal outcomes such as PTB or LBW rates would be most valuable. 

This study found no indication of difference between the uninsured and insured groups in health care service or treatment itself rendered during delivery at the two hospitals. The two groups had similar rates of intrapartum medical interventions, regardless of insurance status. These included fairly high rates of oxytocin augmentation, caesarean section and major resuscitation when compared to published Ontario perinatal statistics [27,28,35,36]. Factors which predisposed the uninsured women to labor dystocia remain unidentified. The use of epidural among uninsured cases was higher than expected (49.7%), even though epidural use and associated patient care is an expensive intervention. 

Both the uninsured and insured groups had higher than expected rates of postpartum hemorrhage (PPH), 3.8% and 4.0% respectively. The PPH rate in the Central East Local Health Integrated Network (CE LHIN) is 30 per 1000 births or 3% [28]. These rates are known to vary from jurisdiction to jurisdiction. Worldwide, the prevalence of PPH is 5% and related to numerous risk factors, the chief of which is related to uterine atony [37]. This incidence of PPH may simply reflect the incidence of PPH in the countries of origin for the immigrants sampled. This would be relevant data to gather in future studies. Further research that explores relationships with socio-demographic variables, such as age, socio-economic status, ethnic background, place of birth, length of time in country, and socio-economic status both within and across health service catchment areas, may help to explain these observed variations. 

A number of the key study findings offer interesting apparent contrasts to the existing perinatal outcomes literature. There were no significant differences in the PTB rates and the LBW rates. However, the overall PTB rate was higher than the Canadian national rate (7.6%), the Ontario provincial rate (7.7%), or the local CE LHIN rate (7.6%). The overall LBW rates were also higher than the Canadian rate (6.0%), the provincial rate (6.1), or the CE LHIN rate (6.3%) [28]. Since both groups had higher PTB and LBW rates than the surrounding regions and general population, these findings could very well be related to the socio-demographic characteristics of residents in the eastern part of the Greater Toronto Area. Shah et al noted, for example, higher rates of LBW and caesarean sections in foreign-born women when compared to Canadian-born women at a large Central Toronto LHIN tertiary health care centre. Moreover, the caesarean section rate increased as the length of residency increased [38]. They noted an increase in the rate of operative delivery in women residing in Canada for five years or more, possibly as a result of increased birth weights and cephalo-pelvic disproportion. Shah et al argue that the etiology is likely multifactorial and possibly explains the loss of the “healthy immigrant effect” [39]. This is a well-documented phenomenon in which new immigrants, selected in part due to their good health status, gradually lose this health advantage over native-born Canadians. They postulate that the reason is due to the adoption of unhealthy habits such as the consumption of fast foods and a more sedentary lifestyle. In a study of foreign-born women with various lengths of residency, Ray et al demonstrated that immigrant women experienced a progressively increasing risk for maternal placental syndromes in pregnancy as their length of residency increased [39]. These syndromes presented as eclampsia, pre-eclampsia, placental abruption and infarction; the risk of other pregnancy complications and chronic diseases may likewise increase over time. The “healthy immigrant effect” is used to explain why the incidence of placental disorders and caesarean sections increase among foreign-born women as their length of residence in Canada increases [39]. This effect may also explain the similarities in some of the perinatal outcomes between our study’s insured and uninsured groups, most notably in terms of medical interventions. Lower birth weights may be tied to ethnic predispositions and, thus, not necessarily reflect any pathology at all. Percentile birth weight curves have not been formally developed for women from different ethno-racial groups [40,41], resulting in possible misclassifications as small for gestational age, with resultant increased testing and parental stress. For these reasons it would be valuable to be able to control for both place of birth and length of residence in future research studies. In addition, using a larger sample size would facilitate deeper exploration of the effect of health care insurance on various perinatal outcomes. Currently, information regarding place of birth and length of Canadian residency is not included in most medical records. 

The ethnic diversity within both the insured and uninsured groups in our study suggests that a large portion of the women were foreign-born. This likelihood is supported by the demographics of the local community, the Central East LHIN. Its total population of 1,419,745 is comprised of 67% immigrants and nonresidents [15]. This may help to explain some of the similarities in perinatal outcomes, most notably in terms of medical interventions, between our study’s insured and uninsured groups. A future population-based study that considers population demographics, community characteristics, as well as access to available resources, would enable exploration of the complex interplay between place of origin, migratory status, health insurance coverage, socio-economic status, available resources, and perinatal outcomes.

### 4.1. Limitations of the Study

The study was limited by aspects of the data source and record retrieval process itself. Retrospective chart reviews are problematic due to inaccuracy and inconsistency in recording [19,22,23]. Key demographic information is often missing, inaccurate, or inconsistently coded. We were unable to match cases on relevant demographic information, such as country of origin, length of residency, and family income, because this information was inconsistently recorded or not recorded at all.

Privacy and confidentiality policies made it difficult to link client data with other sources to obtain income data. (Postal code data provided crude income estimates, but the residential addresses provided by migrant women are often temporary in nature, and this data was not considered useful). Examining or controlling for socio-economic status requires accurate income and educational data. The relatively small geographic region covered in this study is socioeconomically heterogeneous. A correlation between socioeconomic status and migrant health status may exist, and it may be similar to the correlations found between insurance status and the amount of prenatal care or rate of newborn resuscitation. Even if income is proven to have no direct effect on perinatal outcomes, it may remain an important mediating factor. 

The study was also limited by the small sample size that decreased the power of the parametric and non-parametric tests on some of the variables, notably for smoking, APGAR, and mortality. For example, it is possible that the higher smoking rate in the insured group (5.9% *versus* 1.3%), as a known factor for intrauterine growth restriction, may have accounted for the higher SGA rate [29], but the number of smokers was too small to test for a relationship. The small sample size may also help explain the lack of difference in LBW rates between uninsured and insured pregnant women. The rate of smoking during pregnancy for Ontario is 12% [42]. In 2008, the CE Public Health Region reported that 7.0% of their LBW babies were born to smokers, compared to 4.7% for non-smokers [43]. If women in the insured group were Ontario residents for a relatively longer period of time, they may have developed a higher rate of smoking, thereby increasing their rates of PTB and LBW. Controlling for smoking rates would be important in future research using larger sample sizes. Furthermore, accurate analysis of the impact of smoking would have required the amount of smoking to be known, a quantity that was not captured in the provincial antenatal records. The study’s sample size similarly precluded comparative analyses for low APGAR scores at five minutes. APGAR rates were low in both groups (uninsured 1.2%, insured 0.7%), indicating that most of the newborns were healthy babies; the Ontario low APGAR rate is 0.6% [42]. The small sample size also precluded calculations of comparative perinatal mortality rates. 

Future research could attempt to truly isolate the effect of insurance on birth outcomes and control for the other dependent variables with a larger sample size. This is the case when so many variables are presented for analysis. Multiple regression would allow analysis of nominal, dependent variables on the raw scores of birth weight, gestational age, income, ethnicity and other measures of newborn status that could isolate the effect of insurance from the other variables. First, it would be important to determine whether predictors for these variables are highly correlated with each other, precluding regression analysis. Secondly the researcher would need to categorize the dependent and independent variables when (Y = XB + e). Moreover, in the presence of so many nominal variables, the researchers might instead employ odds ratios to more closely examine the risk that lack of insurance poses. 

A prospective study design would enable more reliable collection of mitigating variables; however, vulnerable migrant women with precarious immigration status may be reluctant to participate. A methodological alternative to retrospective record review and data extraction would be the use of health information technology at the point of service provision. A computerized database, such as the Better Outcomes Registry and Network (BORN) Database initiated by the Ontario Ministry of Health and Long Term Care, would greatly facilitate prenatal outcome research by standardizing the documentation of birth indicators and client demographics and facilitating the aggregation and analyses of the resulting data to inform health policy and practice innovations geared at optimal health outcomes [42]. 

In brief, the inherent challenges of data collection via retrospective chart reviews, similarities in ethnic background profiles between the study’s insured and uninsured groups, lack of income data, the inability to determine the length of Canadian residency and thus explore its possible impact on outcomes, together with small sample sizes may have influenced and limited the scope of the study’s findings. The findings are not generalizable since the 175 uninsured mothers and newborn dyads examined represent only a fraction of all uninsured pregnant mothers in the Greater Toronto Area and were not randomly selected. Nonetheless, the findings clearly reveal important differences in prenatal care, while similar care was provided during the births. Additional studies conducted across broader geographic and demographic contexts and with larger sample sizes are clearly needed. 

## 5. Conclusions

This quantitative retrospective study provides level II-2 [43] evidence for health policy analysts and decision-makers. A key goal of equitable health policy should be to address disparity reduction in perinatal care of newcomer populations. The high levels of less-than-adequate and definitively inadequate prenatal care uncovered in this study indicate that uninsured pregnant women experience significantly disparate access to an essential health care service. We also found indications of possible negative effects on medical interventions and neonatal resuscitations. For maternity care providers, high quality healthcare implies healthcare that is integrated, woman-centred, holistic and evidence-based. Early prenatal care remains necessary to optimize perinatal outcomes for migrant mothers and newborns.
